# Mullerian tissue in the rectus abdominis muscle forming an endometrioma: a case report

**DOI:** 10.11604/pamj.2019.34.159.19193

**Published:** 2019-11-25

**Authors:** Yasmeen Akhtar Haseeb

**Affiliations:** 1 Obstetrics and Gynaecology, College of Medicine, Imam Abdulrahman Bin Faisal University, Dammam, Saudi Arabia

**Keywords:** Cesarean scar, endometrioma, rectus muscle

## Abstract

Endometriosis is defined as the presence of endometrial tissue at the sites other than the uterine lining and responds to the cyclical ovarian hormonal activity. It is a multidimensional and a complex disease which has an impact on physical, mental, sexual, and social aspects of the life of a woman. Here is a case report of a patient, presented to the department with pain and swelling in her left side of the previous cesarean scar. It was surgically removed and was diagnosed to be an endometrioma of rectus sheath and rectus abdominis muscle on histopathology.

## Introduction

Endometriosis is a chronic disease. Although, it is seen most commonly in pelvic organs especially the ovaries, rarely it has also been reported in every organ of the female body. Extra pelvic endometrioma is a rare condition. The development of endometrioma in the rectus muscle and rectus sheath is a rare entity and is thought to be due to implantation of the endometrium to this site during pelvis surgeries especially the cesarean sections (CS). Literature shows the prevalence of endometriosis in CS scar to be 0.03 to 0.4% [1,2]. This is a report of a case of endometrioma of rectus abdominis and rectus sheath after 3 previous CS which was excised during myomectomy 6 years after her last CS.

## Patient and observation

**Figure 1:**
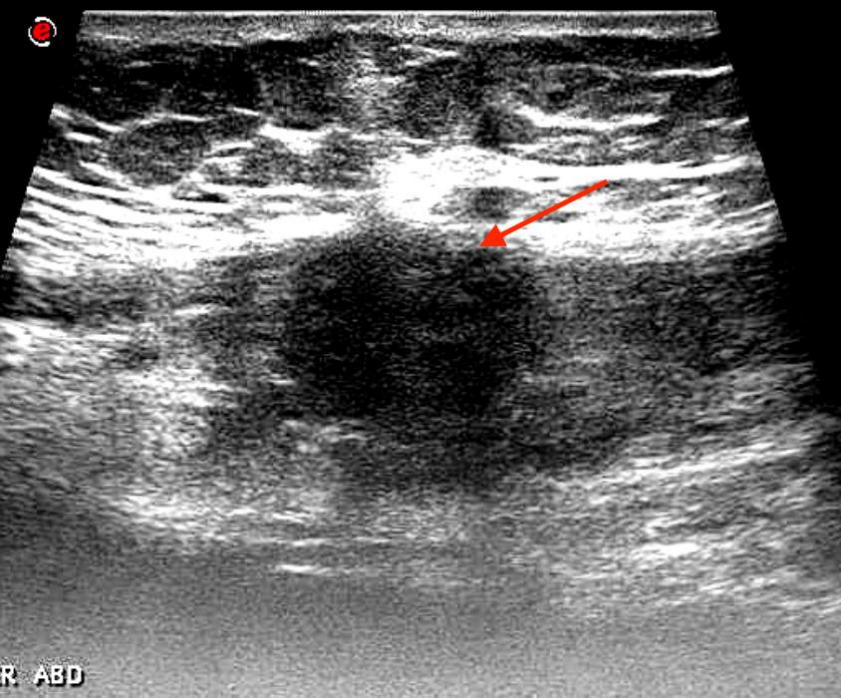
Ultrasound image of the endometrioma on left lower part of rectus muscle

**Figure 2:**
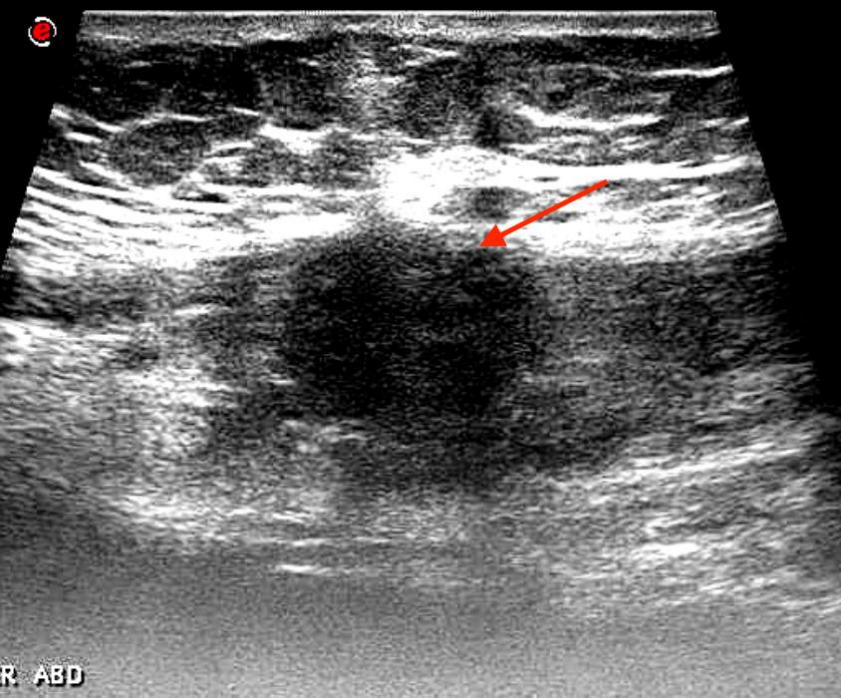
CT scan transverse section of left lower rectus muscle showing endometrioma

A 47 years old lady presented with menorrhagia and lower abdominal pain with a tender swelling in left lower side of previous CS scar. She gave history of lower abdominal pain for the last 4 years with a swelling on left lower part of her cesarean scar. According to the patient she noticed increase in size and pain at least a week before and during menstruation to such an extent that she needed analgesia. She had 3 previous CS and last one was about 6 years ago. Ultrasound showed a mass of 4.1×2.8 cm in lower left side of anterior abdominal wall with minimal blood flow on doppler (Figure 1) and a uterine fibroid of about 8×7 cm in posterior uterine wall. Computerized tomography (CT) scan was arranged for her which showed the same findings in longitudinal and transverse sections, the radiologist gave provisional diagnosis of desmoid, hematoma, lipoma or endometrioma of the rectus muscle or rectus sheath (Figure 2). The patient agreed for myomectomy along with excision of this swelling. During the dissection of anterior abdominal wall, lot of fibrosis was observed with a mass of dark brown colored very adherent to surrounding structures on left rectus sheath involving the rectus muscle as well, it was excised and sent for histopathological analysis, myomectomy was done and no endometriosis was found anywhere in the pelvis. The histopathologist reported it to be an endometrioma of rectus abdominis muscle involving the rectus sheath as well. The patient was discharged after routine post-operative care and she was relieved of her symptoms on follow-up.

## Discussion

**Figure 3:**
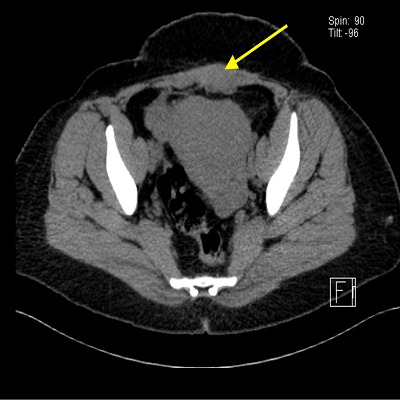
Histopathological picture of endometriosis with endometrial glands, fibromuscular, stroma and blood vessels

Endometriosis was first reported in the ovary by Russel in 1899 [3]. Later on it was reported in almost every organ of the body even in inguinal canal with the suspicion of hernia and on excision it was found to be the endometriosis [4]. Extra pelvic endometriosis is a rare entity. Presence of endometriosis in rectus sheath and rectus muscle in the form of endometrioma is rare. The underlying etiology is thought to be the intraoperative implantation during the CS usually from surgical manipulation [5]. The time of presentation of rectus sheath endometriosis can range from 1 to 24 years with a mean of 4.8 years [6]. Our Patient presented almost 6 years of her last CS. The clinical presentation may range from asymptomatic to functional or nonfunctional swelling in lower abdomen. Examination usually shows a mass of varying size. Ultrasound is a first line diagnostic tool in this regard followed by more invasive investigations like computerized tomograghy (CT) or magnetic resonant imaging techniques (MRI) [7]. The differential diagnosis includes desmoid tumors, fibroma, neuroma or hematoma. Sometimes in rare cases endometriosis of rectus sheath could be a malignant tumor of anterior abdominal wall [8]. The treatment options in such cases include expectant, medical with progesterone, gonadotrophins inhibitors or local excision. Ultrasound guided excision of the endometriosis of rectus abdominis muscle is also an option but needs expertise. This patient was symptomatic, and she was also undergoing myomectomy so excision was done. The definitive diagnosis is always by histopathological analysis. In this patient the diagnosis was also confirmed with histopathology report (Figure 3).

## Conclusion

Endometrioma of rectus sheath and muscle is although rare but can be a source of continuous pain and impact on quality of life of the patient. The surgeons during pelvis surgery should avoid unnecessary manipulation with abdominal packs or roll gauze of other organs and tissues in case of any suspicion, local irrigation with normal saline should be done to avoid such morbidities to the patients.

## Competing interests

The authors declare no competing interests.
